# Divergent modulation of pain and anxiety by GABAergic neurons in the ventrolateral periaqueductal gray and dorsal raphe

**DOI:** 10.1038/s41386-022-01520-0

**Published:** 2022-12-16

**Authors:** Linghua Xie, Hui Wu, Qing Chen, Fang Xu, Hua Li, Qi Xu, Cuicui Jiao, Lihong Sun, Rahim Ullah, Xinzhong Chen

**Affiliations:** 1 Department of Anesthesia, Women’s Hospital, Zhejiang University School of Medicine, Hangzhou, China; 2 Department of Endocrinology, Children’s Hospital of Zhejiang University School of Medicine, National Clinical Research Center for Child Health, Hangzhou, Zhejiang China

**Keywords:** Emotion, Psychology

## Abstract

The ventrolateral periaqueductal gray (vlPAG) collaborates with the dorsal raphe (DR) in pain regulation and emotional response. However, the roles of vlPAG and DR γ-aminobutyric acid (GABA)-ergic neurons in regulating nociception and anxiety are contradictory and poorly understood. Here, we observed that pharmacogenetic co-activation of vlPAG and DR GABAergic (vlPAG-DR^GABA+^) neurons enhanced sensitivity to mechanical stimulation and promoted anxiety-like behavior in naïve mice. Simultaneous inhibition of vlPAG-DR^GABA+^ neurons showed adaptive anti-nociception and anti-anxiety effects on mice with inflammatory pain. Notably, vlPAG^GABA+^ and DR^GABA+^ neurons exhibited opposing effects on the sensitivity to mechanical stimulation in both naïve state and inflammatory pain. In contrast to the role of vlPAG^GABA+^ neurons in pain processing, chemogenetic inhibition and chronic ablation of DR^GABA+^ neurons remarkably promoted nociception while selectively activating DR^GABA+^ neurons ameliorated inflammatory pain. Additionally, utilizing optogenetic technology, we observed that the pronociceptive effect arising from DR^GABA+^ neuronal inhibition was reversed by the systemic administration of morphine. Our results collectively provide new insights into the modulation of pain and anxiety by specific midbrain GABAergic subpopulations, which may provide a basis for cell type-targeted or subregion-targeted therapies for pain management.

## Introduction

In the mammalian brain, the ventrolateral periaqueductal gray (vlPAG) and its neighboring dorsal raphe (DR) nucleus regulate analgesia and anxiety [1, 2]. The vlPAG and DR (vlPAG-DR) consist of a diverse population of neurons, including glutamatergic (Glu), GABAergic, and dopaminergic (DA) neurons. Interestingly, since the DR is embedded in the vlPAG and DR^DA+^ neurons extend laterally into the vlPAG, vlPAG-DR is often regarded as the same area-the ventral PAG (vPAG) [2, 3]. Therefore, the vlPAG-DR is thought to process information cooperatively, especially for pain regulation. It is well established that vlPAG-DR^Glu+^ projections to the rostral ventral medulla (RVM) modulate ascending nociceptive signals and reduce the pain response [4]. Similarly, vlPAG-DR^DA+^ neurons also induce antinociception by co-releasing Glu and DA [1, 5, 6]. ln contrast, vlPAG^GABA+^ but not DR^GABA+^ neurons facilitate nociception by inhibiting the descending pathway (vlPAG-DR^Glu^-RVM) [7]. Although vlPAG^GABA+^ and DR^GABA+^ neurons display opposite roles in controlling feeding behavior [8], the specific function of vlPAG^GABA+^ neurons and DR^GABA+^ neurons in pain regulation remains largely unknown.

It is well documented that vlPAG^GABA+^ neurons act as a central player in endogenous opioid-induced analgesia [9–11]. More precisely, opioids act on the opioid receptors expressed on vlPAG^GABA+^ neurons to inhibit GABA release, which in turn exerts anti-nociceptive effects [12]. Although a study about the analgesic potency of morphine indicates the distinct functions of the vlPAG and DR in pain modulation [13], the involvement of DR^GABA+^ neurons in opioid anti-nociception is still obscure. The analgesic effect induced by intra-DR infusion of morphine, an agonist of opioid receptors, could be reversed by intra-DR infusion of the GABA_A_ receptor antagonist bicuculline [14], which suggests that GABAergic neurons within the vlPAG and DR may play opposite roles in opioid-induced analgesia. It is unclear whether DR^GABA+^ neurons contribute to the analgesic effect of opioid differently from vlPAG^GABA+^ neurons.

In addition to pain regulation, the vlPAG and DR are also involved in anxiety. A higher level of anxiety is observed when activating vlPAG-DR^Glu+^ neurons [6]; however, whether vlPAG^GABA+^ and DR^GABA+^ neurons work cooperatively or independently to integrate anxiety information is controversial. It is reported that local administration of midazolam (a direct GABA_A_ receptor agonist) into the vlPAG enhances the activity of GABAergic neurons but shows no effects on anxiety [15], while intra-DR administration of GABA agonists alleviates cocaine-induced anxiety [16]. However, DR^GABA+^ neuronal activation locally suppresses serotonergic activity, which indirectly elicits anxiety-related behaviors [17]. These studies imply the existence of different regulatory functions in anxiety between vlPAG^GABA+^ and DR^GABA+^ neurons.

Here, we combined cell-type specific chemogenetic manipulations and optogenetic approaches to dissect the function of GABA^+^ neurons within the vlPAG and DR on pain processing and emotional responses. The co-activation of vlPAG-DR^GABA+^ neurons induced hypersensitivity to mechanical stimulation and anxiety-like behavior, while the inhibition of vlPAG-DR^GABA+^ neurons led to anti-nociception and anti-anxiety effects on mice with inflammatory pain. Moreover, we found the opposite effects of separately manipulating vlPAG^GABA+^ and DR^GABA+^ neurons on the nociceptive responses. Additionally, we observed that opioids might not inhibit DR GABA release in the same manner as in the vlPAG. Our results collectively provide new insights regarding the distinct functions of GABA^+^ neurons within the vlPAG and DR in pain and anxiety modulation.

## Materials and methods

### Animals

The GAD2-ires-Cre mice were genotyped according to the protocol provided by the Jackson Laboratory. All procedures were performed following the National Institutes of Health Guidelines for the Care and Use of Laboratory Animals and approved by the Animal Advisory Committee of Zhejiang University.

### Stereotaxic surgeries

Adult male mice anesthetized with isoflurane (1%–3%) in oxygen were mounted in a stereotaxic apparatus (RWD Life Science, Shenzhen, China), and the position of the skull was adjusted horizontally. Then, a small craniotomy was conducted using aseptic techniques, and microinjections were performed with a 10 μL syringe (Hamilton, Nevada, USA) connected to a glass micropipette. Viruses were administered bilaterally in the vlPAG (35–40 nL for all experiments, relative to bregma: ML ± 0.45 mm, AP − 4.70 mm, DV − 3.25 mm) and unilaterally at an angle of 10 degrees in the middle-posterior region of the vlPAG-DR or DR (110 nL for vlPAG-DR, 60 nL for DR; relative to bregma: ML − 0.55 mm, AP − 4.61 mm, DV − 3.16 mm) at a rate of 40 nL/min with a microsyringe pump (KD Scientific, USA).

### Chemogenetic manipulation

Adeno-associated viruses (AAV) carrying fusion genes for Gq-coupled or Gi-coupled designer receptors exclusively activated by designer drugs (Gq DREADD: a modified human M3 muscarinic receptor, hM3Dq; Gi DREADD: a modified human M4 muscarinic receptor, hM4Di) or the control viruses (mCherry) were injected into GAD2-ires-Cre mice. The AAV9-hysn-DIO-hM3Dq/hM4Di-mCherry or AAV9-hysn-DIO-mCherry viruses (Taltool, Shanghai, China) were diluted to approximately 3 × 10^12^ gc/ml before use. Behavioral tests were initiated after a 3-week recovery. Clozapine-N-oxide (CNO; 5 mg/kg; HY17366, MedchemExpress, USA) or vehicle was administered intraperitoneally (i.p.) 30 minutes prior to testing to ensure activation of Gq signaling or Gi signaling in virus-infected GABAergic neurons. Mice were given CNO for each test session with a 2-day interval between tests.

### Optogenetic manipulation

AAV carrying optogenetic inhibitory DIO-eNpHR3.0 vector or control DIO-eYFP vector (Taltool, Shanghai, China) were diluted to approximately 3.12 × 10^12^ gc/ml before use. Optogenetic fiber (diameter 200 μm, Inper, Hangzhou, China) was implanted 0.1 mm above the DR (relative to bregma: ML − 0.55 mm, AP − 4.61 mm, DV − 3.06 mm) and secured to the animal’s skull with dental adhesive cement. For optogenetic manipulation of eNpHR3.0, the optical fiber was connected to yellow light (594 nm, constant), and the laser output was controlled by a Master-8 pulse stimulator and adjusted to 7–8 mW (terminals). During the opioid-related test, morphine sulfate (Mor), a non-selective opioid receptor agonist, was administered (i.p.) at a dose of 1 mg/kg. Thirty minutes after injection, the Von Frey test was conducted under optogenetic inhibition of DR GABAergic neurons.

### Inflammatory Pain Model

Mice were given an injection of 20 μL Complete Freund’s Adjuvant (CFA; Sigma, St. Louis, MO) in the plantar surface of the right hind paw. Pain behavior tests were initiated 3 days or 7 days after paw injections. Anxiety coupled with chronic pain was analyzed 12 days after CFA injection.

### Behavioral testing

#### Von Frey

The Von Frey test was used to measure mechanical nociceptive sensitivity. Mice were put into Plexiglas chambers on a metal wire surface and habituated for 1–2 hours. According to the up-down method described in Bonin et al. [18], nylon monofilaments of forces ranging from 0.008 to 1.4 g were applied to the hind paw (right). Starting from a filament with a mid-range force (0.07 g), if a mouse withdrew the paw, then a filament with descending force (0.04 g) was used for the following stimulation; if the mouse had no response, a filament with ascending force (0.16 g) was used for the following stimulation. A total of six stimuli was allowed for each trial.

### Hargreaves test

The Hargreaves test was used to measure thermal sensitivity to pain. Mice were placed in Plexiglas boxes on a glass surface and habituated for 1–2 hours. Three trials recording the withdrawal latency of the hind paw were conducted with a Hargreaves radiant heat apparatus, and the intensity was set to 20 on the IITC Plantar Analgesia Meter (IITC Life Science, Woodland Hills, CA). The basal paw withdrawal latency in naïve mice was adjusted to 9–12 s, with a cutoff of 20 s to prevent excessive tissue damage.

### Open field test (OFT)

Mice were placed in an open field arena (44 cm × 44 cm × 44 cm) and allowed to explore freely for 12 minutes. The center of the open field was defined as the central 25% of the arena (22 × 22 cm). The location and activity of mice were tracked using an ANY-maze behavioral recording system (Stoelting, USA).

### Elevated Plus Maze (EPM) Test

The apparatus for EPM consisted of two open arms and two closed arms (30 × 5 cm^2^) in an arrangement in which the two arms of each type were opposite to each other. The maze was placed 65 cm above the ground. Each mouse was placed in the center area facing the open arm and allowed to explore freely for 5 minutes. Time spent in the open arms and the number of entries into the open arms were calculated using the video-based ANY-maze system.

### Rotarod test

Animals were exposed to a 5-min habituation session in the rotarod apparatus (LE8205, Panlab, USA) at a slow velocity (4 rpm) facing away from the direction of rotation. In the test session, mice were habituated on the rod for 1 min before the trial, and then the rotarod was set with a start speed of 4 rpm. The acceleration rate of the rotarod was from 4 to 40 rpm over a 5-min period. Each mouse received 3 trials. When the mice fell off the rotating drums, the latency to fall and the acceleration rate of the rotarod were recorded for each trial.

### Home cage activity

Before measurements, the mice were acclimatized to single housing in a cage for 5 days. After CNO injection, the mice were returned to their home cages until the start of the test. Their spontaneous activity in the animal’s home cages was monitored by an ANY-maze behavioral tracking system (Stoelting, USA).

### Immunohistochemistry

Mice were sacrificed 90 min after CNO administration (i.p.) and then perfused with 4% paraformaldehyde (PFA) in PBS. Coronal brain sections (30-μm thickness) were cut by a freezing microtome (CM30503, Leica Biosystems, Germany) and blocked for an hour with blocking solution containing 100% normal donkey serum (NDS; 017–000–121, Jackson ImmunoResearch Laboratories, Inc. USA), 10% bovine serum albumin (BSA; A2153, Sigma‒Aldrich, USA), and 20% Triton X-100 (Sigma‒Aldrich, USA). Then, the sections were incubated with the primary antibody [rabbit anti–c-fos (1:1000; Cell Signaling Technology, CST)] overnight at 4 °C. Sections were incubated with the secondary antibody at room temperature for 1 h [Alexa Fluor 488 anti-rabbit IgG (1:1000; A-21202, Thermo Fisher Scientific)]. Images were captured using an Olympus FV1000 confocal microscope. The number of immune-positive cells was counted and analyzed by ImageJ (NIH, USA).

### In situ hybridization

Mice injected with control or taCaspase3 viruses were perfused with PBS followed by precooled 4% PFA solution, and the brain was postfixed overnight in 4% PFA at 4 °C. In situ hybridization was performed according to the manufacturer’s instructions (RNAscope® multiplex fluorescent manual assay kit, Advanced Cell Diagnostics). Coronal brain sections (18 μm) containing DR were used for in situ hybridization to detect the mRNA expression of *Vgat*.

### Statistical Analyses

Experienced researchers conducted the different experiments independently, and blinded data collectors analyzed the results. All the data are shown as the Mean ± S.E.M, unless otherwise specified. Before analyses, all the data underwent the Kolmogorov–Smirnov normality test. Comparisons between two groups were made using Student’s *t*-test. The variance was found to be similar between the groups as tested using Levene’s test of homogeneity of variances. One-way ANOVA or nonparametric tests (Kruskal‒Wallis test with Dunn’s multiple comparisons test) were used when three or more independent groups were compared. Two-way ANOVA followed by Tukey’s multiple comparisons test was used. As appropriate, a *P* value less than 0.05 was considered to be statistically significant. The analyses were performed by using GraphPad Prism TM 8.0 (version 8.0; GraphPad Software Inc., USA).

## Results

### Co-activation of vlPAG and DR GABAergic (vlPAG-DR^GABA+^) neurons promotes mechanical sensitivity and anxiety

**Fig. 1:**
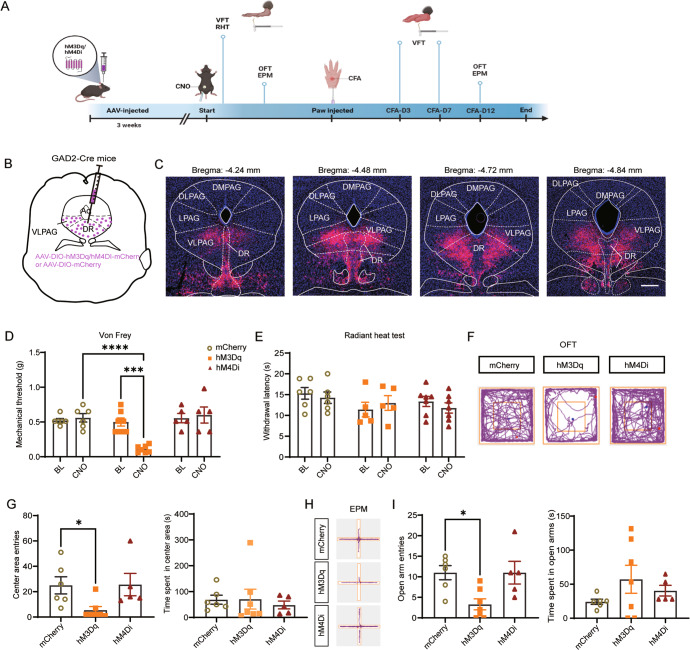
Co-activation of vlPAG-DR^GABA+^ neurons promotes nociceptive sensitivity and anxiety. **A** Experimental timeline and design of the behavioral experiment. **B** Schematic representation of vlPAG-DR injection of Cre-dependent hM3Dq, hM4Di, and mCherry viruses in GAD2-ires-Cre mice. **C** Representative coronal sections of virus infection in both DR and vlPAG from bregma −4.24 to −4.84 mm. **D** Withdrawal threshold of the hind paw in response to Von Frey mechanical stimulation after chemogenetic regulation of vlPAG-DR^GABA+^ neurons following CNO administration. Interaction F(2, 30) = 9.220, *P* = 0.0008; CNO Factor F(1, 30) = 4.312, *P* = 0.0465; Group Factor F(2, 30) = 12.86, *P* < 0.0001. *n* = 6 for mCherry, *n* = 7 for hM3Dq, *n* = 5 for hM4Di. **E** Hind paw withdrawal latency in response to radiant heat after chemogenetic regulation of vlPAG-DR^GABA+^ neurons. Interaction F(2, 30) = 0.6355, *P* = 0.5366; CNO Factor F(1, 30) = 0.08974, *P* = 0.7666; Group Factor F(2, 30) = 1.873, *P* = 0.1712. *n* = 6 for mCherry, *n* = 5 for hM3Dq, *n* = 7 for hM4Di. **F** Representative movement traces in the OFT test showing the locations of mCherry, hM3Dq, and hM4Di groups after CNO administration. **G** Quantitative results of center area entries (left) and the time spent in the center area (right) during the OFT test after activation or inhibition of vlPAG-DR^GABA+^ neurons. Center area entries: *P* = 0.008, Kruskal-Wallis statistic = 8.507; the time spent in the center area: *F* = 0.1686, *P* = 0.8464. *n* = 6 for mCherry, *n* = 7 for hM3Dq, *n* = 5 for hM4Di. **H** Representative movement traces in the EPM test showing the locations of the mCherry, hM3Dq, and hM4Di groups after CNO administration. **I** Quantitative results of open arm entries (left) and the time spent in the open arms (right) of the mCherry, hM3Dq, and hM4Di groups after chemogenetic regulation of vlPAG-DR^GABA+^ neurons following CNO administration. Open arm entries: *F* = 6.053, *P* = 0.0118; the time spent in the open arms: *P* = 0.3099, Kruskal-Wallis statistic = 2.427. *n* = 6 for mCherry, *n* = 7 for hM3Dq, *n* = 5 for hM4Di. BL: baseline. Scale bar, 250 μm. Data are presented as the Mean ± S.E.M. and error bars represent S.E.M. *P < 0.05, ***P < 0.001, ****P < 0.0001.

To evaluate the function of vlPAG-DR^GABA+^ neurons on nociception and anxiety-like behaviors, we designed an experimental timeline (Fig. 1A) and applied an AAV carrying Cre-dependent hM3Dq/hM4Di or mCherry in the vlPAG-DR of GAD2-ires-Cre mice (Fig. 1B). To ensure that the viruses were effective, c-fos staining was performed following CNO stimulation. As expected, compared to the control viruses (mCherry), the number of c-fos positive cells was significantly increased in the hM3Dq group and reduced in the hM4Di group (CNO:mCherry vs. CNO:hM3Dq *P* = 0.0011; CNO:mCherry vs. CNO:hM4Di *P* = 0.0422; Fig. S1A–C). According to the experimental paradigm, the Von Frey filaments assay and radiant heat test were used to measure the sensitivity to mechanical and thermal nociception; the open field test (OFT) and elevated plus maze (EPM) were used to assess anxiety-like behaviors. As observed, the virus infection in the vlPAG-DR on the coronal plane ranged from bregma −4.24 to −4.84 mm (Fig. 1C), and no other subdivisions of the PAG (dorsomedial PAG, dorsolateral PAG, lateral PAG) were infected. Chemogenetic activation of vlPAG-DR^GABA+^ neurons by CNO administration significantly decreased the paw withdrawal threshold in hM3Dq mice compared to mCherry control mice (CNO:mCherry vs. CNO:hM3Dq *P* < 0.0001; Fig. 1D). Furthermore, inhibiting vlPAG-DR^GABA+^ neurons did not influence mechanical sensitivity (CNO:mCherry vs. CNO:hM4Di *P* = 0.9974; Fig. 1D). No significant difference was observed in the mechanical withdrawal threshold in the mCherry, hM3Dq, and hM4Di groups after injection (i.p.) of vehicle (vehicle:mCherry vs. vehicle:hM3Dq *P* = 0.7968; vehicle:mCherry vs. vehicle:hM4Di *P* = 0.4470; Fig. S2A). In addition, neither activation nor inhibition of vlPAG-DR^GABA+^ neurons produced statistically significant changes in the withdrawal latency to radiant heat (CNO:mcherry vs. CNO:hM3Dq *P* = 0.9897; CNO:mcherry vs. CNO:hM4Di *P* = 0.7960; Fig. 1E).

Anxiety is a common comorbidity in patients with chronic pain. Maladaptive and persistent emotional responses to pain incite a vicious cycle that in turn worsens the pain [19, 20]. In addition to the regulation of nociceptive sensitivity, vlPAG-DR neurons are also implicated in anxiety. Therefore, we injected (i.p.) CNO into mCherry-, hM3Dq-, and hM4Di-expressing mice and traced their movements during the behavioral test (Fig. 1F). Compared to the mCherry group, the hM3Dq group showed a decreased number of entries into the center area (CNO:mCherry vs. CNO:hM3Dq *P* = 0.0242; Fig. 1G) in the OFT test, while no significant change in the number of center entries was observed in hM4Di-expressing mice (CNO:mCherry vs. CNO: hM4Di *P* > 0.9999; Fig. 1G). Consistently, hM3Dq mice exhibited fewer entries into the open arms in the EPM test (CNO:mCherry vs. CNO:hM3Dq *P* = 0.0159; Fig. 1H, I). To exclude the impact of CNO on the OFT and EPM tests, we further supplemented the behavior tests in hM3Dq/vehicle and mCherry/vehicle mice. The results showed that there was no significant difference in anxiety-like behavior between hM3Dq/vehicle and mCherry/vehicle groups [(center area entries: *P* = 0.9320; the time spent in the center area: *P* = 0.7384); (open arm entries: *P* = 0.4404; the time spent in the open arms: *P* = 0.6672); Fig. S2B, C]. According to the movement trace of the hM3Dq/CNO group, it seemed that co-activating the vlPAG-DR^GABA^ neurons might inhibit locomotor activity. Indeed, the difference in locomotor activity could confound emotional measures. Therefore, we next examined motor ability using the rotarod test and no significant difference was observed in the latency to fall off the rotarod or the rotational velocity at the time of falling between the mCherry/CNO and hM3Dq/CNO mice (latency: *P* = 0.6615; rotational velocity: *P* = 0.4792; Fig. S3A). Additionally, we assessed the physical activity of mice in their home cage after CNO administration. When exposed to the familiar and safe home cage, the hM3Dq mice showed no significant difference in locomotor activity compared with the mCherry mice (CNO:mCherry vs. CNO:hM3Dq *P* = 0.9438; Fig. S3B). Therefore, we suggest that the decrease in exploratory activity during the OFT or EPM test might be due to the extremely anxious phenotype of hM3Dq/CNO mice. Taken together, our results demonstrated that chemogenetic activation of vlPAG-DR^GABA+^ neurons produced hyperalgesia and anxiety-like behavior in naïve mice.

### Enhanced vlPAG^GABA+^ signaling results in the potentiation of mechanical sensitivity

**Fig. 2:**
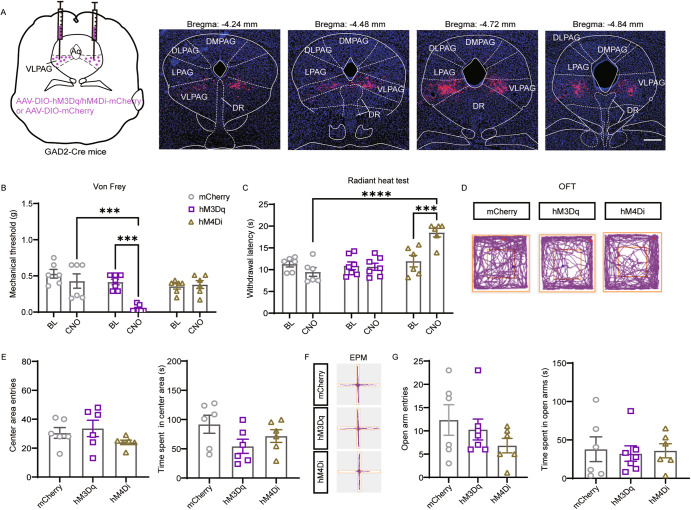
Enhanced vlPAG^GABA+^ signaling results in the potentiation of mechanical sensitivity. **A** Schematic representation of vlPAG injection of hM3Dq, hM4Di, and mCherry viruses in GAD2-ires-Cre mice and representative coronal sections of virus infection in the vlPAG from bregma −4.24 to −4.84 mm. **B** Withdrawal threshold of the hind paw in response to Von Frey mechanical stimulation after chemogenetic regulation of vlPAG^GABA+^ neurons following CNO administration. Interaction F(2, 32) = 6.932, *P* = 0.0032; CNO Factor F(1, 32) = 10.88, *P* = 0.0024; Group F(2, 32) = 10.47, *P* = 0.0003. *n* = 6 for mCherry, *n* = 7 for hM3Dq, *n* = 6 for hM4Di. **C** Hind paw withdrawal latency in response to radiant heat after chemogenetic regulation of vlPAG^GABA+^ neurons. Interaction F(2, 32) = 10.40, *P* = 0.0003; CNO Factor F(1, 32) = 3.736, *P* = 0.0621; Group Factor F(2, 32) = 15.35, *P* < 0.0001. *n* = 6 for mCherry, *n* = 7 for hM3Dq, *n* = 6 for hM4Di. **D** Representative movement traces in the OFT test showing the locations of the mCherry, hM3Dq, and hM4Di groups after CNO administration. **E** Quantitative results of center area entries (left) and the time spent in the center area (right) during the OFT test after activation or inhibition of vlPAG^GABA+^ neurons. Center area entries: *P* = 0.2570, Kruskal-Wallis statistic = 2.795; the time spent in the center area: *F* = 2.126, *P* = 0.1539. *n* = 6 for mCherry, hM3Dq and hM4Di. **F** Representative movement traces in the EPM test showing the locations of the mCherry, hM3Dq, and hM4Di groups after CNO administration. **G** Quantitative results of open arm entries (left) and the time spent in the open arms (right) of the mCherry, hM3Dq, and hM4Di groups after chemogenetic regulation of vlPAG^GABA+^ neurons following CNO administration. Open arm entries: *P* = 0.4542, Kruskal-Wallis statistic = 1.648; the time spent in the open arms: *F* = 0.05965, *P* = 0.9423. *n* = 6 for mCherry, *n* = 7 for hM3Dq, *n* = 6 for hM4Di. Scale bar, 250 μm. Data are presented as the Mean ± S.E.M. ***P < 0.001, ****P < 0.0001.

According to the GABA disinhibition hypothesis, vlPAG^GABA+^ neurons, as an essential part of the descending nociceptive neural pathway, are commonly considered interneurons that locally inhibit the analgesic effect of vlPAG^Glu+^ neurons [21]. To investigate the explicit role of vlPAG^GABA+^ neurons, we bilaterally injected the Cre-dependent hM3Dq, hM4Di, and control viruses into the vlPAG at extremely low rates to ensure restricted expression in the vlPAG (Fig. 2A). Compared to the mCherry group, CNO-triggered activation of vlPAG^GABA+^ neurons in hM3Dq-expressing mice produced a significant decrease in the paw withdrawal threshold in response to mechanical stimulation (CNO:mCherry vs. CNO:hM3Dq *P* = 0.0003; Fig. 2B). Interestingly, the inhibition of vlPAG^GABA+^ neurons in hM4Di-expressing mice had no significant influence on the paw withdrawal threshold in response to mechanical stimulation (CNO:mCherry vs. CNO:hM4Di *P* = 0.9899; Fig. 2B). However, it induced a remarkable increase in the withdrawal latency to thermal stimulation compared to control mice (CNO:mCherry vs. CNO:hM4Di *P* < 0.0001; Fig. 2C).

It has been reported that the activation of PAG/DR^Glu+^ neurons facilitates anxiety-like behavior [6], but the influence of vlPAG^GABA+^ neurons on anxiety remains unclear. According to our results, no significant difference was observed among these three groups in terms of the center area entries (CNO:mCherry vs. CNO:hM3Dq *P* > 0.9999; CNO:mCherry vs. CNO:hM4Di *P* = 0.6066) and the time spent in the center area (CNO:mCherry vs. CNO:hM3Dq *P* = 0.1322; CNO:hM4Di *P* = 0.5281) in the OFT test after CNO administration (Fig. 2D, E). The EPM test also showed no significant difference among these three groups [(open arm entries: mCherry vs. hM3Dq *P* > 0.9999; mCherry vs. hM4Di, *P* = 0.4927); (time spent in open arms: mCherry vs. hM3Dq *P* = 0.9392; mCherry vs. hM4Di *P* = 0.9928); Fig. 2F, G]. Together, our results collectively demonstrate that activation of vlPAG^GABA+^ neurons causes hypersensitivity to mechanical stimulation; however, their inhibition decreases the sensitivity to noxious heat only.

### Chemogenetic inhibition or ablation of DR^GABA+^ neurons increases mechanical nociceptive sensitivity

**Fig. 3:**
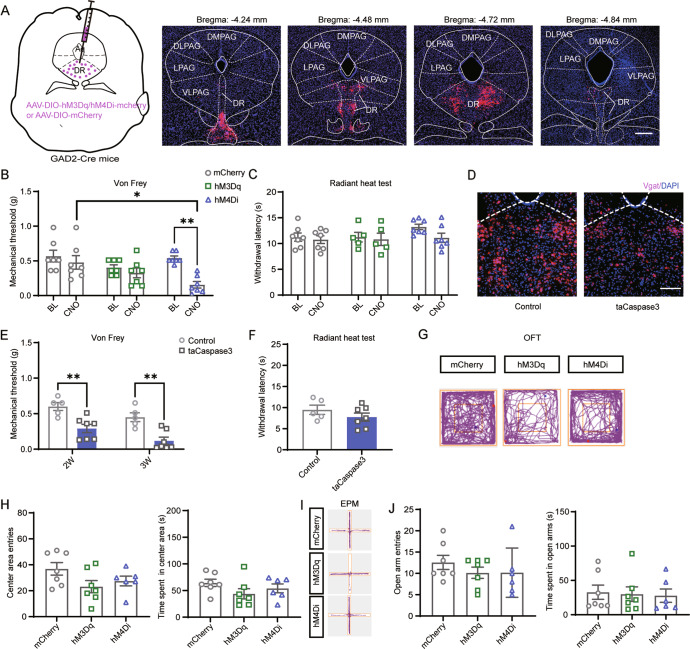
Chemogenetic inhibition or ablation of DR^GABA+^ neurons increases mechanical nociceptive sensitivity. **A** Schematic representation of DR injection of hM3Dq, hM4Di, and mCherry viruses in GAD2-ires-Cre mice and representative coronal sections of virus infection in the DR from bregma −4.24 to −4.84 mm. Scale bar, 250 μm. **B** Withdrawal threshold of the hind paw in response to Von Frey mechanical stimulation after chemogenetic regulation of DR^GABA+^ neurons following CNO administration. Interaction F(2, 36) = 3.453, *P* = 0.0425; CNO Factor F(1, 36) = 11.59, *P* = 0.0016; Group Factor F(2, 36) = 4.279, *P* = 0.0215. *n* = 7 for mCherry, hM3Dq and hM4Di. **C** Hind paw withdrawal latency in response to radiant heat after chemogenetic regulation of DR^GABA+^ neurons. **D** Representative images of *Vgat-*positive neurons (red) in the DR 3 weeks after control or taCaspase3 virus injection. Scale bar 100 μm. **E** Decreased mechanical threshold over time after ablation of DR^GABA+^ neurons by taCaspase3. Interaction F(1, 10) = 0.05683, *P* = 0.8164; Time Factor F(1, 10) = 11.34, *P* = 0.0072; Group Factor F(1, 10) = 23.58, *P* = 0.0007. *n* = 5 for control, *n* = 7 for taCaspase3. **F** Effect of taCaspase3 virus injection for 3 weeks on thermal withdrawal latency. *P* = 0.2611, t(10) = 1.191. *n* = 5 for Control, *n* = 7 for taCaspase3. **G** Representative movement traces in the OFT test showing the locations of the mCherry, hM3Dq, and hM4Di groups after CNO administration. **H** Quantitative results of center area entries (left) and the time spent in the center area (right) during the OFT test after the activation or inhibition of DR^GABA+^ neurons. Center area entries: *F* = 2.490, *P* = 0.1127; the time spent in the center area: *F* = 1.590, *P* = 0.2327. *n* = 7 for mCherry and hM3Dq, *n* = 6 for hM4Di. **I** Representative movement traces in the EPM test showing the locations of the mCherry, hM3Dq, and hM4Di groups after CNO administration. **J** Quantitative results of open arm entries (left) and the time spent in the open arms (right) of the mCherry, hM3Dq, and hM4Di groups after chemogenetic regulation of DR^GABA+^ neurons following CNO administration. Open arm entries: *F* = 0.6361, *P* = 0.5415; the time spent in the open arms: *F* = 0.05862, *P* = 0.9433. *n* = 7 for mCherry and hM3Dq, *n* = 6 for hM4Di. 2 W: 2 weeks; 3 W: 3 weeks. Data are presented as the Mean ± S.E.M. *P < 0.05, **P < 0.01.

The DR is reported to provide a substantial proportion of serotonergic innervation to the forebrain and is implicated in pain and emotional responses; however, the contribution of DR^GABA+^ neurons to these responses remains largely uncharacterized. To explore the specific role of DR^GABA^ neurons, we injected the Cre-dependent hM3Dq, hM4Di, and control viruses into the DR (Fig. 3A). Our results showed that chemogenetic activation of DR^GABA+^ neurons did not affect the withdrawal threshold in response to mechanical stimulation in naïve mice (CNO:mCherry vs. CNO:hM3Dq *P* = 0.5915; Fig. 3B). However, CNO administration markedly reduced the mechanical threshold in mice injected with hM4Di virus (CNO:mCherry vs. CNO:hM4Di *P* = 0.0166; Fig. 3B). However, neither activation nor inhibition of DR^GABA+^ neurons had a pronounced effect on the withdrawal latency in response to radiant heat (CNO:mCherry vs. CNO:hM3Dq*P* > 0.9999; CNO:mCherry vs. CNO:hM4Di *P* > 0.9999; Fig. 3C). According to our results, the role of DR^GABA+^ neurons in nociceptive perception was completely opposite of vlPAG-DR^GABA+^ or vlPAG^GABA+^ neurons. Therefore, to further confirm our results, we ablated GABAergic neurons by injecting AAV-DIO-taCaspase3 viruses into the DR. To confirm GABAergic neuronal ablation, we performed in situ hybridization to analyze the mRNA expression of the vesicular GABA transporter (Vgat; a marker of GABAergic neurons). As expected, *Vgat* mRNA in the DR was markedly diminished three weeks after taCaspase3 injection (Fig. 3D). Consistent with the results obtained from hM4Di-mediated inhibition, ablation of DR^GABA+^ neurons resulted in a dramatic decrease in mechanical threshold (2 W:Control vs. 2 W:taCaspase3 *P* = 0.0023; 3 W:Control vs. 3 W:taCaspase3 *P* = 0.0012; Fig. 3E), but did not influence the threshold to thermal stimulation (Control vs. taCaspase3 *P* = 0.2611; Fig. 3F). The administration of CNO showed no apparent effect on anxiety levels in all groups during the OFT [(center area entries: mCherry vs. hM3Dq *P* = 0.0774; mCherry vs. hM4Di *P* = 0.2851); (time spent in center area: mCherry vs. hM3Dq *P* = 0.1613; mCherry vs. hM4Di *P* = 0.6117); Fig. 3G, H] and EPM tests [(open arm entries: mCherry vs. hM3Dq *P* = 0.5222; mCherry vs. hM4Di *P* = 0.5528); (time spent in the open arms: mCherry vs. hM3Dq *P* = 0.9718; mCherry vs. hM4Di *P* = 0.9206); Fig. 3I, J]. Our data demonstrate that specific inhibition of DR^GABA+^ neurons causes mechanical hypersensitivity; however, the activation of these neurons has no obvious effect on anxiety-like behavior during naïve states.

### Inhibition of vlPAG-DR^GABA+^ or activation of DR^GABA+^ neurons attenuates mechanical hypersensitivity in inflammatory pain

**Fig. 4:**
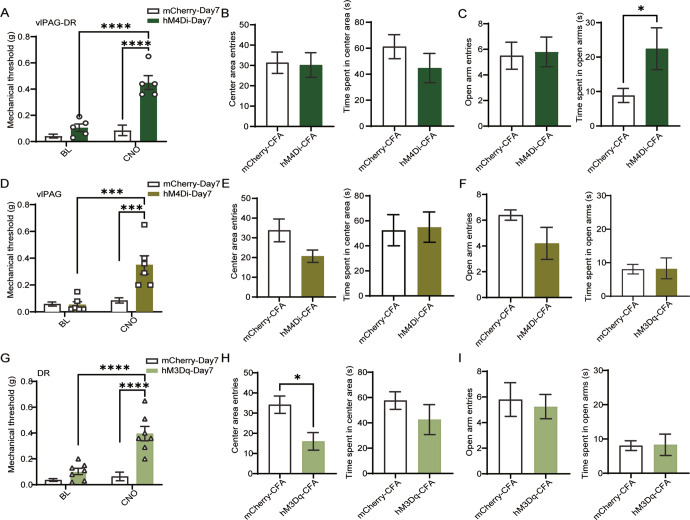
Inhibition of vlPAG-DR^GABA+^ or activation of DR^GABA+^ neurons attenuates nociceptive hypersensitivity in inflammatory pain. **A** Mechanical withdrawal threshold after chemogenetic inhibition of vlPAG-DR^GABA+^ neurons on the seventh day after CFA exposure. Interaction F(1, 18) = 17.53, *P* = 0.0006; CNO Factor F(1, 18) = 29.10, *P* < 0.0001; Group Factor F(1, 18) = 35.75, *P* < 0.0001. *n* = 6 for mCherry, *n* = 5 for hM4Di. **B**, **C** Quantitative results of center area entries and the time spent in the center area during the OFT test (**B**) and open arm entries and the time spent in the open arms during the EPM test (**C**) after chemogenetic manipulation of vlPAG-DR^GABA+^ neurons in mCherry and hM4Di mice injected with CFA. Center area entries: *P* = 0.8902, t(9) = 0.1420; the time spent in the center area: *P* = 0.2826, t(9) = 1.143. Open arm entries: *P* = 0.8525, t(9) = 0.1914; the time spent in the open arms: *P* = 0.0480, t(9) = 2.287. *n* = 6 for mCherry, *n* = 5 for hM4Di. **D** Mechanical withdrawal threshold after chemogenetic suppression of vlPAG^GABA+^ neurons on the seventh day after CFA injection. Interaction F(1, 20) = 2.63, *P* = 0.0020; CNO Factor F(1, 20) = 18.11, *P* = 0.0004; Group Factor F(1, 20) = 11.71, *P* = 0.0027. *n* = 6 for mCherry and hM4Di. **E**, **F** Quantitative results of center area entries and time spent in the center area during the OFT test (**E**) and open arm entries and the time spent in the open arms during the EPM test (**F**) after chemogenetic manipulation of vlPAG^GABA+^ neurons in mCherry and hM4Di mice injected with CFA. Center area entries: *P* = 0.0656, t(9) = 2.096; the time spent in the center area: *P* = 0.8896, t(9) = 0.1429. *n* = 5 for mCherry, n = 6 for hM4Di. Open arm entries: *P* = 0.2063, U = 5.5; the time spent in the open arms: *P* = 0.3049, t(8) = 1.096. *n* = 5 for mCherry and hM4Di. **G** Mechanical withdrawal threshold after chemogenetic activation of DR^GABA+^ neurons on the seventh day after CFA injection. Interaction F(1, 26) = 15.33, *P* = 0.0006; CNO Factor F(1, 26) = 22.35, *P* < 0.0001; Group Factor F(1, 26) = 34.59, *P* < 0.0001. *n* = 8 for mCherry, *n* = 7 hM3Dq. **H**, **I** Quantitative results of center area entries and the time spent in the center area during the OFT test (**H**) and open arm entries and the time spent in the open arms during the EPM test (**I**) after chemogenetic manipulation of DR^GABA+^ neurons in mCherry and hM3Dq mice injected with CFA. Center area entries: *P* = 0.0166, t(9) = 2.937; the time spent in the center area: *P* = 0.4061, t(9) = 0.8716. *n* = 6 for mCherry, *n* = 5 hM3Dq. Open arm entries: *P* = 0.8254, *U* = 9; the time spent in the open arms: *P* = 0.6511, t(8) = 0.4697. *n* = 5 for mCherry, *n* = 4 hM3Dq. Data are presented as the Mean ± S.E.M. *P < 0.05, ***P < 0.001, ****P < 0.0001.

Since we observed that the amplified activity of vlPAG-DR^GABA+^ neurons was responsible for nociception, we wondered whether inhibiting these neurons could alleviate the phenotype of inflammatory pain. To prove our hypothesis, we investigated the effect of vlPAG-DR^GABA+^ neuronal inhibition on a CFA inflammatory pain model. Mechanical hyperalgesia induced by CFA administration for seven days was relieved by the suppression of vlPAG-DR^GABA+^ neurons in hM4Di-expressing mice following CNO administration (CNO:mCherry-Day7 vs. CNO:hM4Di-Day7 *P* < 0.0001; Fig. 4A). In addition, the anti-nociceptive effect of CNO in hM4Di-expressing mice was also observed on the third day after CFA administration (CNO:mCherry-Day3 vs. CNO:hM4Di-Day3 *P* = 0.0007; Fig. S4A). It is well known that chronic pain leads to anxiety-like behaviors [22]. Therefore, we performed the OFT and EPM tests 12 days after CFA injection to evaluate anxiety-like behavior. We found that inhibiting vlPAG-DR^GABA+^ neurons did not affect center entries or central time in the OFT (center entries: mCherry-CFA vs. hM4Di-CFA *P* = 0.8902; time spent in the center area: mCherry-CFA vs. hM4Di-CFA *P* = 0.2826; Fig. 4B); however, it increased the time spent in the open arms, indicating the potential anti-anxiety effect of vlPAG-DR^GABA+^ neuronal inhibition (open arm time: mCherry-CFA vs. hM4Di-CFA *P* = 0.0480; Fig. 4C).

Previous studies found that microinjection of GABA antagonists or inhibiting vlPAG^GABA+^ neurons has a significant anti-nociceptive effect against noxious stimuli [10, 21]. Therefore, we further investigated the role of vlPAG^GABA+^ neurons in CFA-induced inflammatory pain. The hM4Di-induced GABAergic suppression in the vlPAG attenuated mechanical hyperalgesia on the seventh day after CFA injection (CNO:mCherry-Day7 vs. CNO:hM4Di-Day7 *P* = 0.0004; Fig. 4D) and moderately increased the mechanical withdrawal threshold on the third day after CFA treatment (CNO:mcherry-Day3 vs. CNO:hM4Di-Day3 *P* = 0.0252; Fig. S4B). Subsequently, OFT and EPM tests were performed to detect whether inhibiting vlPAG^GABA+^ neurons would affect anxiety-like behavior. Compared to mCherry mice, hM4Di-expressing mice with CNO administration showed a mild reduction in center entries in the OFT test (center entries: mCherry-CFA vs. hM4Di-CFA *P* = 0.0656; Fig. 4E) and decreased entries into the open arms in the EPM test (open arm entries: mCherry-CFA vs. hM4Di-CFA *P* = 0.2063; Fig. 4F); however, these effects did not reach statistical significance. The above results suggest that the suppression of vlPAG^GABA+^ neurons mainly plays an analgesic role in CFA-induced inflammatory pain.

We next examined whether activating DR^GABA+^ neurons alleviated mechanical hyperalgesia in a CFA model. Our results showed that activating DR^GABA+^ neurons relieved hyperalgesia on the seventh day after CFA injection (CNO:mCherry-Day7 vs. CNO:hM3Dq-Day7 *P* < 0.0001; Fig. 4G) but had no improvement in the mechanical threshold on the third day after CFA injection (CNO:mCherry-Day3 vs. CNO:hM3Dq-Day3 *P* = 0.7750; Fig. S4C). Moreover, compared to mCherry mice, hM3Dq-expressing mice injected with CNO displayed a decrease in center area entries (mCherry-CFA vs. hM3Dq-CFA *P* = 0.0166; Fig. 4H). However, no significant difference was observed in EPM test (open arm entries: hM3Dq-CFA vs. mCherry-CFA *P* = 0.8254; time spent in the open arms: hM3Dq-CFA vs. mCherry-CFA *P* = 0.6511; Fig. 4I). The above results suggest that activating DR^GABA+^ neurons mainly plays an analgesic role in CFA-induced inflammatory pain and promotes anxiety-like behaviors.

### Morphine relieves hyperalgesia triggered by photoinhibition of DR^GABA+^ neurons

**Fig. 5:**
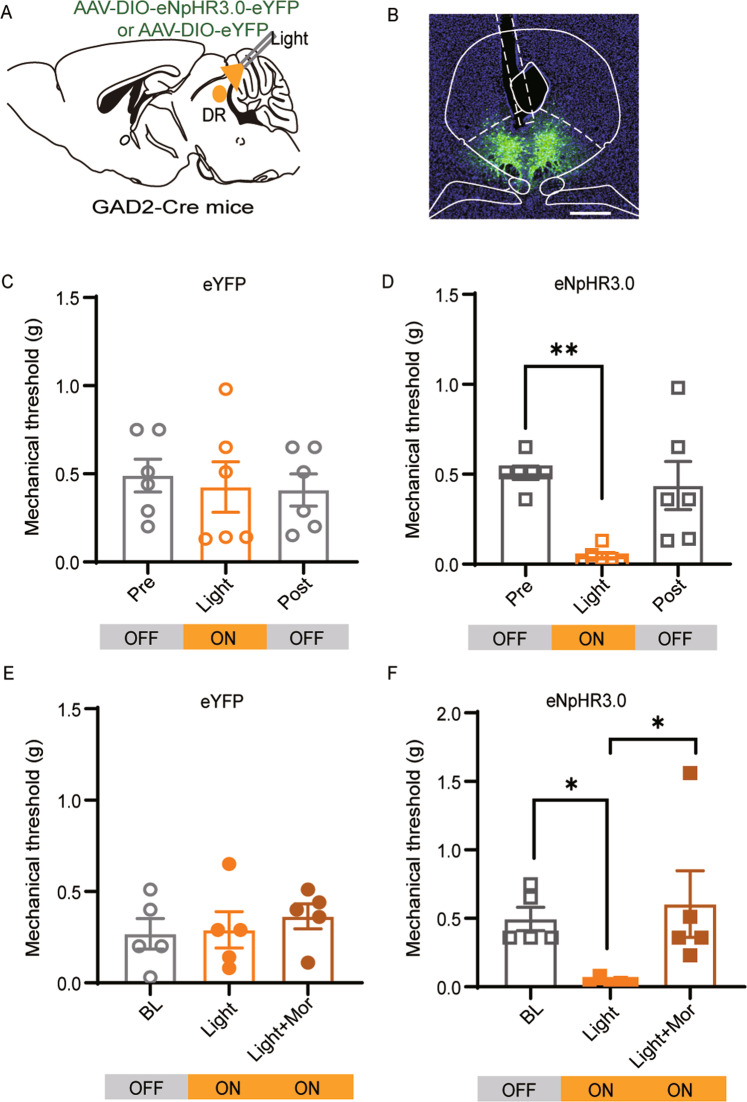
Morphine reverses the mechanical hyperalgesia induced by optogenetic inhibition of DR^GABA+^ neurons. **A** Schematic representation of AAV-DIO-eNpHR3.0-eYFP or AAV-DIO-eYFP injection into the DR of GAD2-ires-cre mice. **B** Representative micrograph of virus infection and optic fiber implantation site in the DR. **C**, **D** Mechanical withdrawal threshold of the eYFP (**C**) and eNpHR3.0 groups (**D**) following yellow light stimulation (594 nm, constant). eYFP: *F* = 0.1485, *P* = 0.8633; eNpHR3.0: *P* = 0.0003, Kruskal-Wallis statistic = 11.66. *n* = 6 for eYFP, *n* = 6 for eNpHR3.0. **E**, **F** Effect of systemic administration of morphine (Mor, 1 mg/kg) on the mechanical hypersensitivity induced by optogenetic inhibition of DR^GABA+^ neurons. eYFP-Mor: *F* = 0.3521, *P* = 0.7102; eNpHR3.0-Mor: *P* = 0.0014; Kruskal-Wallis statistic = 9.842.*n* = 5 for eYFP-Mor, *n* = 5 for eNpHR3.0-Mor. Mor Morphine. Scale bar, 250 μm. Data are presented as the Mean ± S.E.M. *P < 0.05, **P < 0.01.

It is well established that opioids inhibit vlPAG^GABA+^ neurons to produce an anti-nociceptive effect [12]. According to our data described above, inhibiting DR^GABA+^ neurons produced robust mechanical hyperalgesia. To better understand the functions of vlPAG^GABA+^ and DR^GABA+^ neurons in pain regulation, we applied an optogenetic strategy to assess the role of DR^GABA+^ neurons in opioid-mediated antinociception. The Cre-dependent AAV viruses expressing eYFP with or without eNpHR3.0 were injected into the DR of GAD2-ires-Cre mice, and optical fibers were implanted above the DR (Fig. 5A, B). Consistent with the results of chemogenetic inhibition, optogenetic suppression of DR^GABA+^ neurons produced a significant decrease in the mechanical threshold in eNpHR3.0-expressing mice (BL vs. Light *P* = 0.0026; Fig. 5D) but not in eYFP-expressing mice (BL vs. Light *P* = 0.9119; Fig. 5C). However, systemic administration of morphine relieved the hyperalgesia triggered by photoinhibition of DR^GABA+^ neurons (eNpHR3.0: BL vs. Light *P* = 0.0145; eNpHR3.0: Light vs. Light+Mor *P* = 0.0278; Fig. 5E, F). These data suggest that hyperalgesia induced by the photoinhibition of DR^GABA+^ neurons could be reversed by systemic administration of morphine.

## Discussion

In the present study, we found that co-activation of vlPAG-DR^GABA+^ neurons was responsible for the development of mechanical hypersensitivity and negative emotional responses such as anxiety in naïve mice, while inhibiting these neurons induced anti-nociception and anti-anxiety effects on CFA-induced inflammatory pain. Similarly, the activation of vlPAG^GABA+^ but not DR^GABA+^ neurons potentiated sensitivity to mechanical stimulation. However, surprisingly, chemogenetic inhibition or ablation of DR^GABA+^ neurons promoted sensitivity to mechanical stimulation in naïve mice. Interestingly, the specific inhibition of vlPAG^GABA+^ neurons or activation of DR^GABA+^ neurons attenuated the hypersensitivity to mechanical stimulation induced by CFA injection. Furthermore, systemic administration of morphine dramatically attenuated the pro-nociceptive effects of inactivating DR^GABA+^ neurons. These results provide new insights into the different role of GABAergic neurons within the vlPAG and DR in the distinct modulation of nociception and anxiety.

Consistent with a previous study reporting that vlPAG^GABA+^ interneurons exerted an inhibitory effect on the vlPAG^Glu^-RVM, facilitating nociception [7], we found that co-activation of vlPAG-DR^GABA+^ neurons or selective activation of vlPAG^GABA+^ neurons caused mechanical hyperalgesia. Furthermore, the suppression of vlPAG-DR^GABA+^ or specific vlPAG^GABA+^ neurons effectively attenuated mechanical hypersensitivity in a CFA model. The anatomical and functional homogeneity of the vlPAG and DR [2, 3] indicate that activating DR^GABA+^ neurons is also expected to exacerbate nociception. However, according to our observation, inhibition and chronic ablation instead of activation of DR^GABA+^ neurons increased mechanical sensitivity to nociception. Previous studies have mainly focused on DR^GABA+^ neurons serving as interneurons to locally inhibit 5-HT neurons, which produce hyperalgesia [17]. However, recent tracing research reveals that vlPAG^GABA+^ instead of DR^GABA+^ interneurons send a presynaptic input to DR serotonergic neurons [23]. These results indicate that DR^GABA+^ neurons involved in pain modulation are independent of DR serotonergic systems, while almost no related research has been reported. Future research can focus on DR^GABA+^ projecting neurons, as some pain-related brain regions (such as the nuclei of the stria terminalis and central amygdala) are strongly innervated by DR^GABA+^ output neurons [24]. Additionally, the DR also receives massive GABAergic projections from the vlPAG [23, 25], we speculate that the activation of vlPAG^GABA+^ neurons might inhibit the activity of the DR via feedforward inhibition, which may be a possible explanation for the hyperalgesia induced by vlPAG-DR^GABA+^ or vlPAG^GABA+^ neuronal activation. Overall, the opposite effects of vlPAG-DR^GABA+^ and DR^GABA+^ neurons on pain modulation may be ascribed to a balance for the dynamic network activity between vlPAG and DR GABAergic neurons. Therefore, future work should be focused on investigating whether and how vlPAG^GABA+^ neurons play a dominant role in the modulation of pain when simultaneously modulating vlPAG-DR^GABA+^ neuronal activities.

The vlPAG and DR are potentially critical sites for the anti-nociceptive actions of opioids [26]. Opioid receptors are densely distributed throughout vlPAG^GABA+^ interneurons but are primarily distributed in the presynaptic afferents of DR^GABA+^ neurons [27]. The difference in receptor distribution suggests that opioids may exert analgesic effects in vlPAG ^GABA+^ and DRN ^GABA+^ neurons via distinct mechanisms. A previous study has established that opioids inhibit GABA release in the vlPAG to produce anti-nociceptive effects [28]. Our results that chemogenetic inhibition of vlPAG^GABA+^ neurons alleviates mechanical hyperalgesia in CFA-treated mice are consistent with the above study. Similarly, another study also indicates that morphine triggers the release of 5-HT in the DR and speculated that 5-HT indirectly inhibits DR^GABA+^ neurons to alleviate nociceptive sensitivity [14]. However, this speculation seemed to conflict with our study that instead of producing analgesia, inhibiting DR^GABA+^ neurons significantly increased the sensitivity to mechanical stimulation, and morphine treatment reversed the mechanical hyperalgesia induced by photoinhibition of DR^GABA+^ neurons. Interestingly, a recent study using in vivo electrophysiological recordings showed that morphine directly activated 5-HT neurons by increasing the firing rate of putative 5-HT neurons without affecting DR^GABA+^ neurons [29]. Activation of the 5-HT system has been suggested as a useful strategy for the treatment of pain and comorbid chronic pain-related depression [30, 31]. These data suggest that morphine-triggered 5-HT release may account for the anti-nociceptive effects of morphine on the hyperalgesia induced by DR^GABA+^ neuronal inhibition. Our results indicate that vlPAG^GABA+^ and DR^GABA+^ neurons exert distinct roles in pain modulation and that morphine may produce its analgesic effects in the vlPAG and DR via different mechanisms.

The activation of PAG-DR^Glu^ neurons produces high indices of anxiety [6], and we hypothesize that inhibiting GABAergic neurons contributes to elevated anxiety-like behavior by indirectly activating Glu neurons within the vlPAG and DR. However, we found that inhibiting vlPAG^GABA+^ neurons did not affect anxiety-related behavior. Consistent with our results, a recent study illustrated that photoinhibition of vlPAG^GABA+^ neurons did not produce anxiety-like behavior or aversion in naïve mice [32]. Contrary to our results, Lowery-Gionta showed that inhibition of vPAG^GABA+^ neurons promoted anxiety, which was due to the disinhibition of serotonin neurons [3]. However, it is important to note that the enhanced activity of 5-HT neurons was reported to alleviate anxiety and affective disorders. Therefore, vPAG^GABA^ activation rather than inhibition is hypothesized to indirectly suppress serotonin neurons to elicit anxiety-related behaviors [2, 33, 34].

In conclusion, we found that activation and inhibition of vlPAG-DR^GABA+^ neurons bidirectionally regulate nociception and anxiety-like behaviors. We also found that vlPAG^GABA+^ and DR^GABA+^ neurons play different roles in modulating the sensitivity to mechanical stimuli in both naïve and inflammatory pain mice.

## Supplementary information

Supplementary figure
