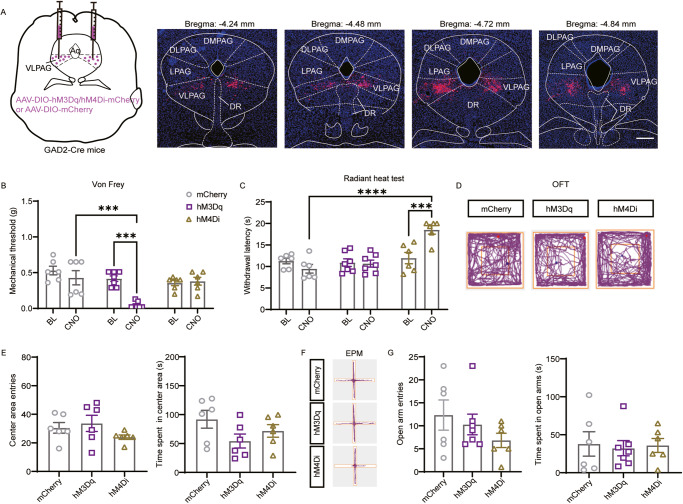# Correction: Divergent modulation of pain and anxiety by GABAergic neurons in the ventrolateral periaqueductal gray and dorsal raphe

**DOI:** 10.1038/s41386-023-01654-9

**Published:** 2023-07-19

**Authors:** Linghua Xie, Hui Wu, Qing Chen, Fang Xu, Hua Li, Qi Xu, Cuicui Jiao, Lihong Sun, Rahim Ullah, Xinzhong Chen

**Affiliations:** 1 Department of Anesthesia, Women’s Hospital, Zhejiang University School of Medicine, Hangzhou, China; 2 Department of Endocrinology, Children’s Hospital of Zhejiang University School of Medicine, National Clinical Research Center for Child Health, Hangzhou, Zhejiang China

**Keywords:** Emotion, Psychology

Correction to: *Neuropsychopharmacology* 10.1038/s41386-022-01520-0, published online 16 December 2022

In the original article, the 4th image of Fig. 2A was inadvertently an incorrect version and has now been replaced. This error does not affect the conclusions of the article. The original article has been corrected.