# Supplementary material for: Divergent modulation of pain and anxiety by GABAergic neurons in the ventrolateral periaqueductal gray and dorsal raphe
Source: Neuropsychopharmacology. 2022 Dec 16;48(10):1509–19. doi: 10.1038/s41386-022-01520-0 (PMC10425368; doi:10.1038/s41386-022-01520-0)

Supplementary Figure 1

(A) Representative images of c-fos staining in mCherry, hM3Dq, and hM4Di groups after CNO injection to verify virus potency. Scale bar 100 μm.

(B) Quantitative results of c-fos positive cells in mCherry, hM3Dq, and hM4Di groups after CNO injection. hM3Dq: P=0.0011, t(9)=4.705; hM4Di: P=0.0422, t(8)=2.415. n=5 for mCherry, n= 6 for hM3Dq (3B, upper); n=5 for mCherry and hM4Di (3B, down).

(C) Quantitative results of the number of c-fos positive cells colocalized with viruses after CNO injection. hM3Dq: P<0.0001 t(9)=8.547; hM4Di: P=0.0004, t(9)=5.509. n=5 for mCherry, n=6 for hM3Dq and hM4Di.

Scale bar 100 μm. Data are presented as Mean ± S.E.M. * P<0.05, ** P<0.01, *** P<0.001, **** P<0.0001.

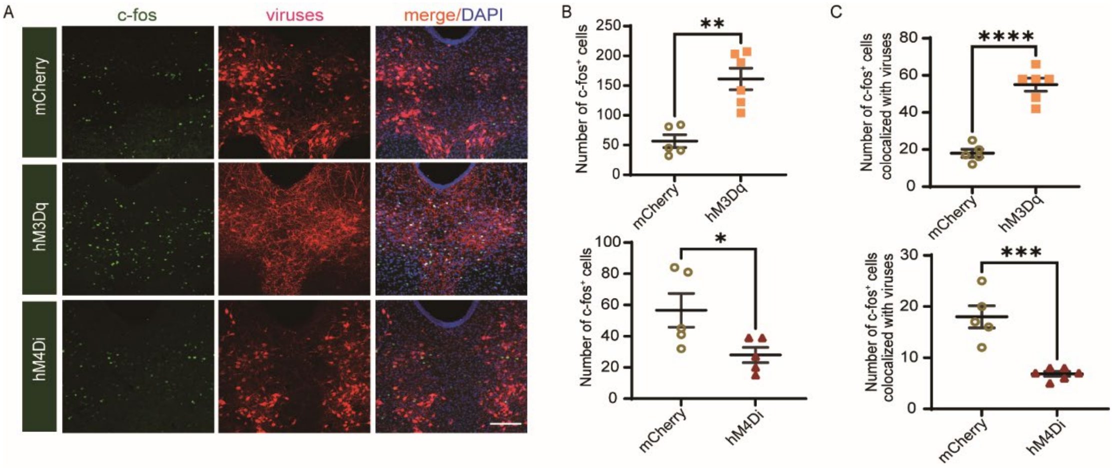

Supplementary Figure 2

(A) Mechanical withdrawal threshold of the hind paw in response to Von Frey mechanical stimulation after the injection of vehicle in mCherry, hM3Dq, and hM4Di groups. Interaction F(2, 36)=0.7431, P=0.4828; vehicle factor F(1, 36)=0.1389, P=0.7115; Group factor F(2, 36)=1.224, P=0.3061. n=7 for mCherry, hM3Dq, hM4Di.

(B) Quantitative results of center area entries (left) and the time spent in the center area (right) during the OFT test after injection of vehicle in mCherry and hM3Dq groups. Center area entries: P=0.9320, t(10)=0.08750; the time spent in the center area: P=0.7384, t(10)=0.3435. n=6 for mCherry and hM3Dq.

(C) Quantitative results of open arm entries (left) and the time spent in the open arms (right) after the injection of vehicle (i.p) in mCherry and hM3Dq groups. Open arm entries: P=0.4404, t(10)=0.8034; the time spent in the open arms: P=0.6672, t(10)=0.4430. n=6 for mCherry and hM3Dq.

BL: baseline; veh: vehicle. Data are presented as Mean ± S.E.M.

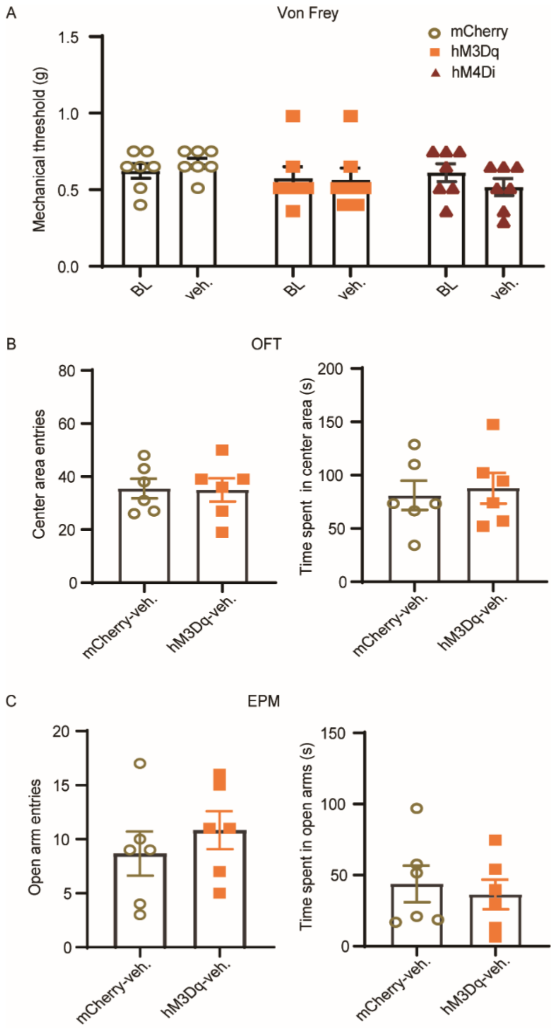

Supplementary Figure 3

(A) The latency to fall off the rotating drums (left) and mean rotational velocity at the time of falling (right) after CNO administration in mCherry and hM3Dq groups.

Latency: Interaction $F(1, 22)= 0.3673$, $P=0.5507$; CNO Factor $F(1, 22)=1.856$, $P=0.1869$; Group Factor $F(1, 22)=1.050$, $P=0.3166$.

Velocity: Interaction $F(1, 22)=0.6063$, $P=0.4445$; CNO Factor $F(1, 22) =1.601$, $P=0.2190$; Group Factor $F(1, 22)=1.642$, $P=0.2134$. n=6 for mCherry, n=7 for hM3Dq.

(B) Quantitative results of total distance in the home cage after CNO administration in mCherry and hM3Dq groups. $P=0.9438$, $t(11)= 0.07214$. n=7 for mCherry, n=6 for hM3Dq.

Data are presented as Mean ± S.E.M.

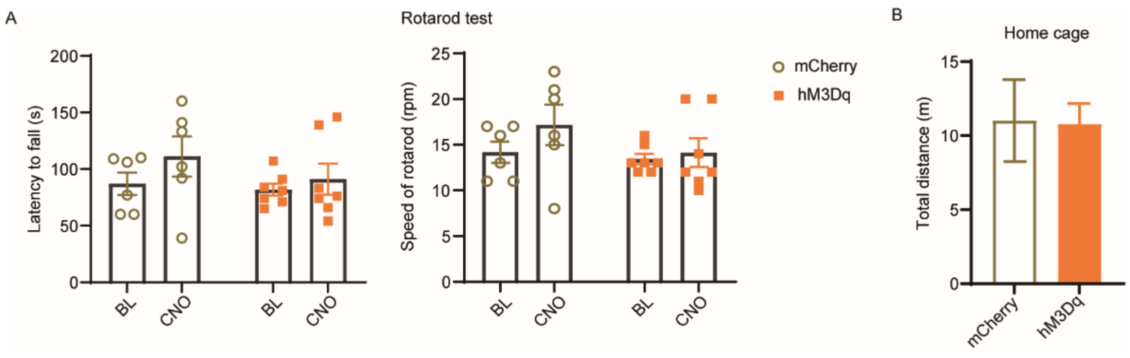

Supplementary Figure 4

(A) Mechanical withdrawal threshold after chemogenetic inhibition of vlPAG-DR^{GABA+} neurons on the third day after CFA exposure.

Interaction F(1, 9)=7.494, *P*=0.0229; CNO Factor F(1, 9)=5.557, *P*=0.0428; Group Factor F(1, 9)=12.70; *P*=0.0061. n=6 for mCherry, n=5 for hM4Di.

(B) Mechanical withdrawal threshold after chemogenetic suppression of vlPAG^{GABA+} neurons on the third day after CFA exposure.

Interaction F(1, 20)=4.589, *P*=0.0447; CNO Factor F(1, 20)=1.724, *P*=0.2040; Group Factor F(1, 20)=5.850, *P*=0.0252. n=6 for mCherry and hM4Di.

(C) Mechanical withdrawal threshold after chemogenetic activation of DR^{GABA+} neurons on the third day after CFA exposure.

Interaction F(1, 18)=0.7313, *P*=0.4037; CNO Factor F(1, 18)=0.03188; *P*=0.8603; Group Factor F(1, 18)=0.2481, *P*=0.6245. n=6 for mCherry, n=5 for hM3Dq.

Data are presented as Mean ± S.E.M. * *P*<0.05, *** *P*<0.001.

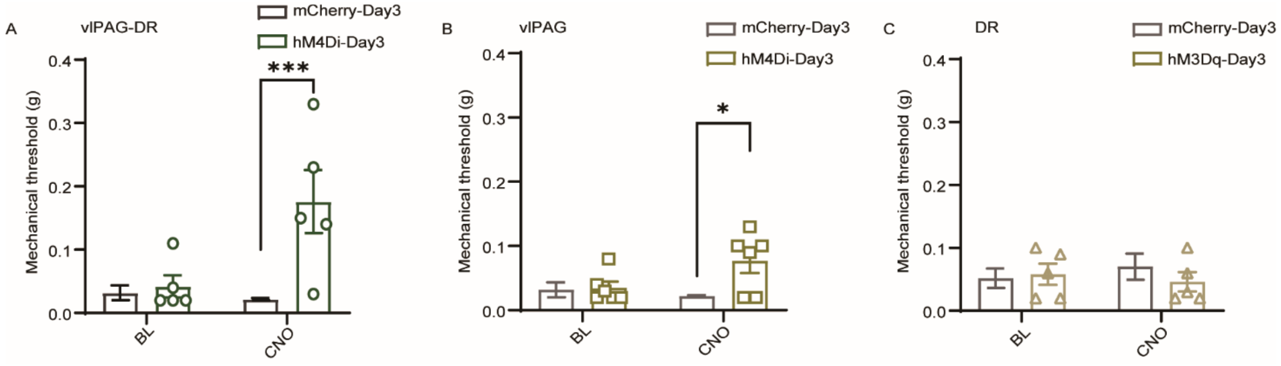

Supplement: Supplementary file 1 — Supplementary figure [file 41386_2022_1520_MOESM1_ESM.pdf]
